# Unexpected discovery of multiple myeloma following cardiomyopathy

**DOI:** 10.1002/ccr3.1297

**Published:** 2017-11-28

**Authors:** Souhail Dahraoui, Jean Uwingabiye, Badia Belarj, Asmae Biaz, Achraf Rachid, Abdellah Dami, Sanae Bouhsain, Zohra Ouzzif, Samira Elmachatni Idrissi

**Affiliations:** ^1^ Department of Clinical Biochemistry and Toxicology Mohammed V Military Teaching Hospital Faculty of Medicine and Pharmacy Mohammed V University Rabat Morocco

**Keywords:** Anemia, cardiorenal syndrome, multiple myeloma

## Abstract

We report the case of multiple myeloma of unexpected discovery in an old patient admitted to the emergency department of cardiomyopathy. This observation emphasizes the need for exploring any anemia before linking it to heart failure or kidney disease. Serum protein electrophoresis remains crucial especially in the elderly patients.

## Introduction

Multiple myeloma (MM) is a hematological malignancy characterized by the proliferation of monoclonal plasma cells in the bone marrow 1. Anemia is present in two‐thirds of patients with MM 2. Triad of chronic kidney disease, heart failure, and anemia has been described in clinical practice 3. The association of the MM with the cardiorenal syndrome can pose a diagnostic problem. We report the case of immunoglobulin G lambda MM of unexpected discovery following acute decompensated heart failure.

## Case Report

An 80‐year‐old male patient, with history of chronic anemia refractory to martial treatment and treated with erythropoietin on an outpatient basis, admitted to the cardiology department of the Mohammed V Military Hospital for the management of decompensated heart failure.

The onset of the symptomatology dates back to 3 months of admission by the installation of dyspnea associated with orthopnea and edemas of the lower limbs of the rapid deterioration requiring hospitalization in our institution.

The clinical examination completed by the Doppler echocardiography showed the dilated cardiomyopathy with apical necrosis, severe aortic insufficiency, and mitral insufficiency requiring valvular replacement.

Laboratory tests revealed serum creatinine level at 141.6 *μ*mol/L (*N*: 53–115) with estimated glomerular filtration rate using the modification of diet in renal disease (MDRD) study equation at 42 mL/min/1.73 m^2^, C‐reactive protein (CRP) at 6.9 g/L (*N*: <5.0 mg/L), the sedimentation rate was 55 mm at the first hour, and lactate dehydrogenase (LDH) was normal at 234 IU/L (*N* < 243).

The plasma albuminemia was 33.69 g/L (*N*: 35–50), protidemia was 57 g/L (*N*: 64–83), and calcium was normal: serum calcium corrected with albumin at 93 mg/L (*N*: 90–100). Proteinuria was 0.56 g/24 h.

The remainder of the biochemical assessment was unremarkable. In addition, the blood count showed the normochromic normocytic anemia (hemoglobin = 8.3 g/dL) without abnormality of other bloodlines.

**Figure 1:**
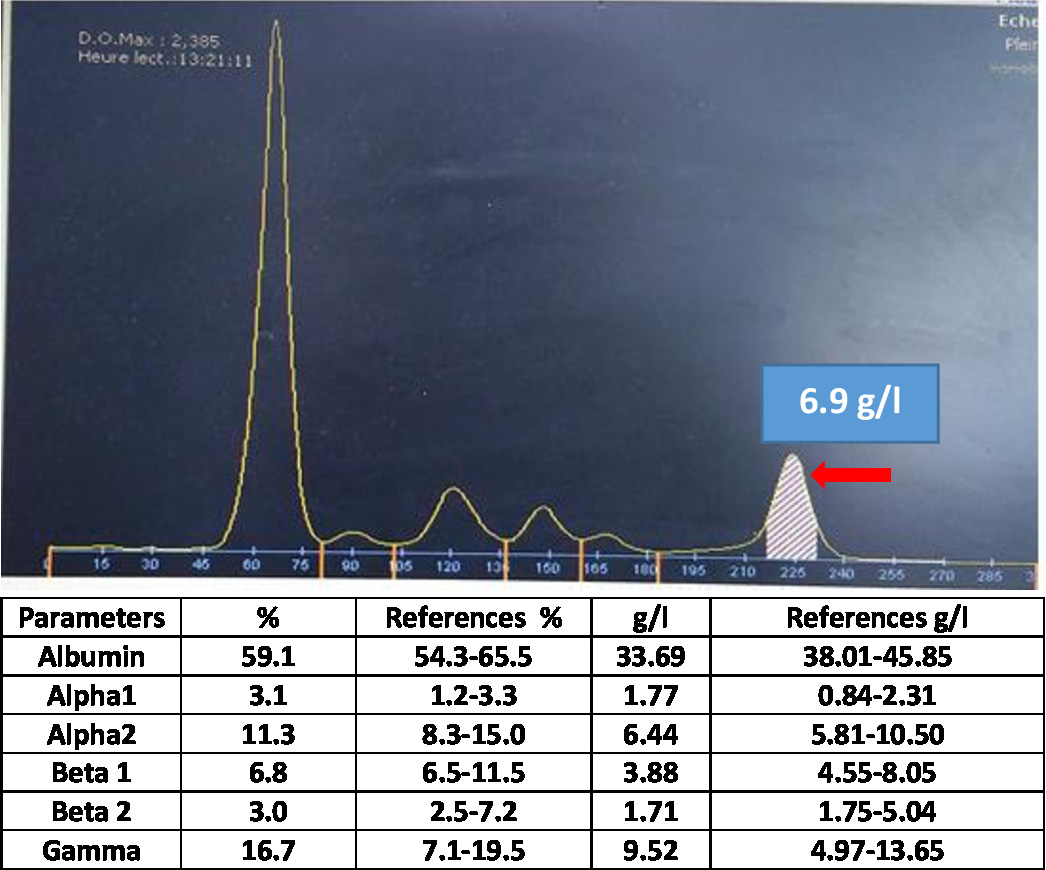
Serum protein electrophoresis showing the presence of a monoclonal peak migrating in the gamma globulin area amounted at 6.9 g/L.

**Figure 2:**
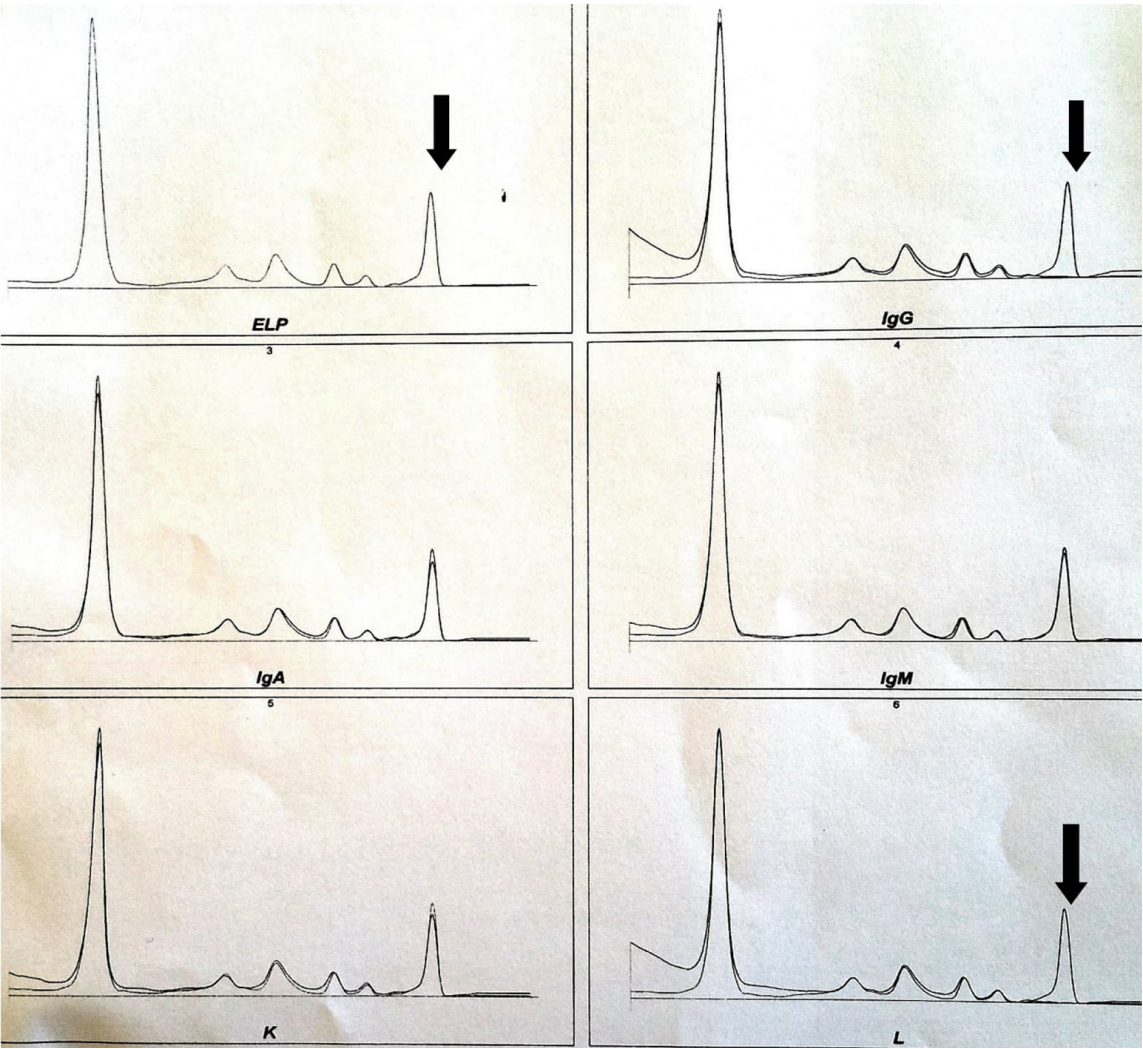
The immunotyping of monoclonal proteins by immunosubtraction showing the presence of the IgG lambda monoclonal immunoglobulin.

**Figure 3:**
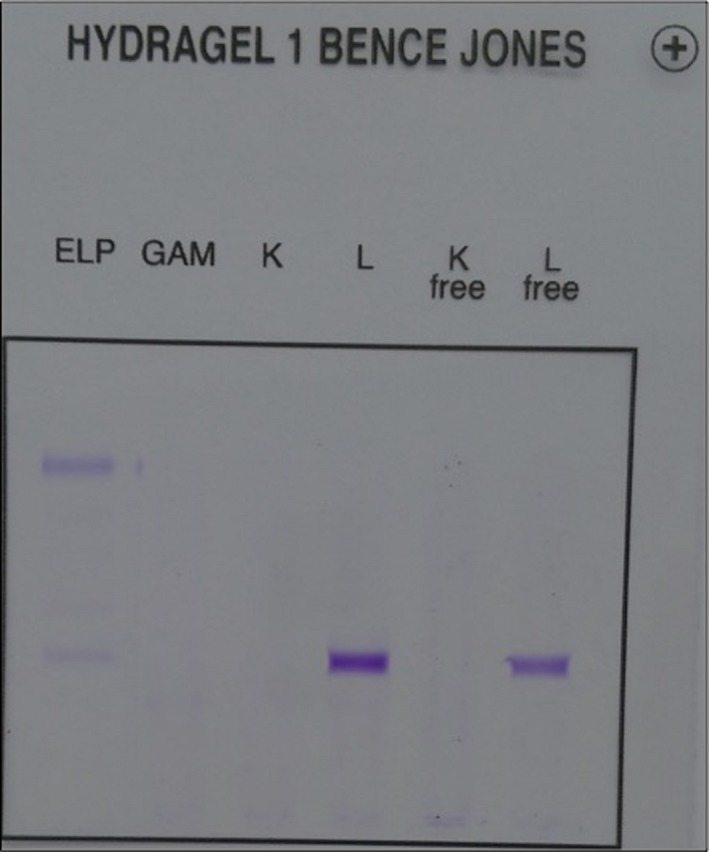
The immunofixation urine test showing lambda free light chain isotypes.

Serum protein electrophoresis demonstrated the presence of a monoclonal peak migrating in the gamma globulin area amounted at 6.9 g/L (Fig. 1). Immunotyping of monoclonal proteins by immunosubtraction (CAPILARYS Sebia) showed the presence of IgG lambda monoclonal immunoglobulin (Fig. 2). Immunofixation urine test revealed the lambda free light chains (Fig. 3).

A complete radiological assessment showed the absence of osteolytic lesions.

Given these results, the bone marrow examination has objectified a rich marrow invaded up to 24% by the dystrophic plasma cells. The patient was referred to the Clinical Hematology Department for further treatment.

## Discussion

This 80‐year‐old male patient, on arrival at the hospital emergency room, presented an evocative symptom of acute decompensated heart failure; apart from the renal insufficiency and anemia found during the intake assessment in the cardiology department, no clinical or biological symptoms of myeloma could be evoked; the biochemical and hematological assessments at the arrival were little contributory factors.

Moreover, the anemia is by far the most common hematological pathology in the elderly and it is a common diagnostic and therapeutic problem in clinical practice. The prevalence of anemia increases with age for both sexes regardless of the threshold values used for hemoglobin and the criteria for inclusion of subjects 4.

The definition of the World Health Organization remains usable: An elderly subject is anemic when hemoglobin (Hb) is <12 g/dL for women and <13 g/dL for men 5.

The heart failure is a public health problem, and its incidence is steadily increasing. This probably reflects the aging of the population, as well as improved treatments for heart disease, high blood pressure, and other cardiovascular risk factors 3.

Anemia is a common disorder in heart failure, and it is very prevalent in patients with heart failure than in the general population 6. The causes of anemia in the heart failure are diverse: There is both hemodilution and decreased production of red blood cells due to iron deficiency, renal insufficiency, inflammation, intake of the conversion enzyme inhibitor, and decrease in factors activating the production of erythropoietin 7, 8.

In these patients with heart failure, a decrease in renal function is frequently found. There are multiple causes of renal insufficiency in cardiac insufficiency: hypovolemia, nephrotic drugs, prostatic obstruction/hydronephrosis, and renin–angiotensin–aldosterone system blockers 9.

The chronic renal failure leads to erythropoietin deficiency anemia at an advanced stage.

However, the anemia can settle earlier in the evolution. A further cause, such as iron deficiency, folate deficiency, or vitamin B12 deficiency, should be investigated before treatment with erythropoietin.

The cardiorenal syndrome is therefore a clinical and physiopathological entity demonstrating the close relationship between the kidney and the heart. In addition, anemia is one of the common factors in heart and kidney disease. The heart failure can be the cause or the aggravation of both anemia and chronic kidney disease. Chronic kidney disease, may also be the cause or the aggravation of both anemia and heart failure. There is therefore a vicious circle between these three diseases, causing or aggravating each other 7.

In our case report, the cardiorenal syndrome could explain the profound anemia, but it could mainly result from the medullary insufficiency linked to the infiltration of the bone marrow by malignant plasma cells.

Indeed, the multiple myeloma is one of the most common diseases affecting the elderly. The average age of onset in the population is 72 years 10.

Anemia is present in two‐thirds of patients with MM 2. Its etiopathogenesis is multifactorial: medullary plasma cell infiltration, cytokine‐induced erythropoiesis suppression, hemodilution linked to the hyperprotidemia, myeloablative chemotherapy, amyloidosis, iron and vitamin deficiency, and decreased secretion of erythropoietin in patients with renal insufficiency 2.

Through this observation, we emphasize the need for exploring any anemia before starting treatment and before linking it to the cardiorenal syndrome, especially in the elderly. Indeed, the iterative treatment by the erythropoietin, which has been administrated to the patient in ambulatory, has undoubtedly contributed to this misdiagnosis.

The serum protein electrophoresis is an integral part of the assessment of acute or chronic renal failure. It can contribute to the etiologic diagnosis by screening for monoclonal gammopathies frequently complicated with renal insufficiency, such as multiple myeloma, or to explore a possible inflammatory syndrome associated with it. The results of the assessment in this patient led us to suggest the resumption of laboratory tests for diagnostic and etiological purposes. For these reasons, serum protein electrophoresis was requested and has demonstrated hypoprotidemia with the presence of a monoclonal peak migrating in the area of gamma globulin amounted at 6.9 g/L. These results pushed us to carry out an immunosubtraction which showed the presence of IgG lambda monoclonal immunoglobulin. Furthermore, immunofixation urine test revealed free lambda immunoglobulin light chains.

The bone marrow examination is the decisive step in the diagnostic process of MM, and it helps to highlight the abnormal plasma cell infiltration quantitatively and qualitatively. The bone marrow plasma cells ≥10% is an important criterion for diagnosis of MM. Indeed, an evolution of the diagnostic criteria was proposed in 2014 by the International Myeloma Working Group (IMWG), thus making it possible to distinguish MM, indolent MM, and monoclonal gammopathies of undetermined significance (MGUS) 11.

The bone lesions in MM often dominate the clinical picture. There was no evidence of bone lesions in our patient and among its medical history, no pathological fracture was found. According to the IMWG diagnostic criteria, the appearance of more than one focal lesion on magnetic resonance imaging associated with medullary plasmacytosis greater than 10% is a new diagnostic criterion for MM. In our case, the radiological assessment was based on standard radiographs only.

Multiple myeloma is an incurable hemopathy, but the appearance of new therapeutics has improved the management. The therapeutic choice is adapted to age, complications of myeloma, prognosis, and cytogenetic study of plasma cells. The median survival does not exceed 5–7 years, but the prognosis varies between patients: Some die within a few months and others survive longer than 10 years 12.

## Conclusion

There is a close link between heart failure, anemia, and chronic kidney disease, one of which can cause or aggravate the other. This case illustrates the importance of exploring any anemia before linking it to heart failure or kidney disease. Indeed, serum protein electrophoresis remains essential especially in the elderly even in the absence of evocative clinical signs allowing the diagnosis of myeloma and accelerating the therapeutic management.

## Authorship

S.D., J.U., and S.E.I.: were involved in collecting patient's data and in literature searching, and wrote also the manuscript. B.B., A.B., A.R., A.D., S.B., and Z.O.: participated in literature search, collected the pictures, and revised the manuscript. All authors read and approved the final version of manuscript.

## Conflict of Interest

None declared.
